# Developments in statistical inference when assessing spatiotemporal disease clustering with the tau statistic

**DOI:** 10.1016/j.spasta.2020.100438

**Published:** 2021-04

**Authors:** Timothy M. Pollington, Michael J. Tildesley, T. Déirdre Hollingsworth, Lloyd A.C. Chapman

**Affiliations:** a MathSys CDT, University of Warwick, UK; b Zeeman Institute (SBIDER), School of Life Sciences, and Mathematics Institute, University of Warwick, UK; c Big Data Institute, Li Ka Shing Centre for Health Information and Discovery, University of Oxford, UK; d London School of Hygiene & Tropical Medicine, UK

**Keywords:** Second order dependence, Pointwise confidence interval, Bias corrected accelerated BCa, Percentile confidence interval, Spatial bootstrap, Graphical hypothesis test

## Abstract

The *tau statistic*τ uses geolocation and, usually, symptom onset time to assess global spatiotemporal clustering from epidemiological data. We test different methods that could bias the clustering range estimate based on the statistic or affect its apparent precision, by comparison with a baseline analysis of an open access measles dataset.

From re-analysing this data we find evidence against no clustering and no inhibition, p-value∈[0,0⋅022] (global envelope test). We develop a tau-specific modification of the Loh & Stein spatial bootstrap sampling method, which gives bootstrap tau estimates with 24% lower sampling error and a 110% higher estimated clustering endpoint than previously published (61⋅0 m *vs.* 29 m) and an equivalent increase in the clustering area of elevated disease odds by 342%. These differences could have important consequences for control efforts.

Correct practice of graphical hypothesis testing of no clustering and clustering range estimation of the tau statistic are illustrated in the online Graphical abstract. We advocate proper implementation of this useful statistic, ultimately to reduce inaccuracies in control policy decisions made during disease clustering analysis.

## Highlights

• Point estimation methods can heavily bias disease clustering range estimates.
• A 110% radial clustering bias is amplified to 342% on an areal intervention scale.
• A modified Loh & Stein spatial bootstrap loses less unique pair information.
• Clustering endpoint estimates appear more precise using this modified bootstrap.
• BCa CIs not percentile CIs are preferred for asymmetric bootstrap distributions.

## Graphical abstract

**Figure:**
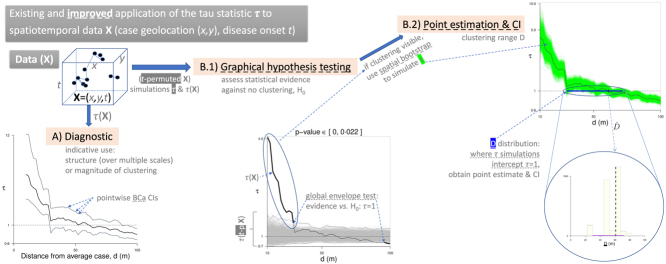

## 1 Introduction

Assessing if *spatiotemporal clustering* is present and measuring its magnitude and range is informative for epidemiologists working to control infectious diseases. The *tau statistic* (Section 2) is more appropriate than most statistics for this task as it measures spatiotemporal rather than just spatial clustering, produces non-parametric estimates (without process assumptions) and, unlike the K function (Gabriel and Diggle, 2009), offers a relative magnitude in the difference of risk, rate or odds of disease versus the background level (Section 2.1) (Lessler et al., 2016, Pollington et al., 2019a). The tau statistic herein should not be confused with ‘Kendall’s tau statistic/rank correlation coefficient’ (Bland, 2000). This study is motivated by a review of its use that found that its current implementation inflates type I errors (incorrectly rejecting a true null hypothesis) when testing for no clustering and no inhibition, and may bias estimates of the range of clustering (Pollington et al., 2019a).

We investigate these aspects by analysing a well-studied open access measles dataset containing household geolocations and symptom onset times of cases (Section 3.1). It represents a *spatially discrete process* since infection is only recorded and can only occur at discrete household locations, so the (statistical) support is not spatially continuous (Diggle et al., 2010).

We adopt an ordered approach: we first test for no clustering and no inhibition (Section 3.3) and then, conditional on finding evidence against this null hypothesis, we estimate the clustering endpoint and its first sampling error estimate (Section 3.4; online Graphical abstract). This approach is contrary to the current methods applied to the tau statistic and similar statistics (Pollington et al., 2019a), which incorrectly combine graphical hypothesis testing for no clustering and estimation of the clustering range (Section 3.2). We hope these improved methods will encourage proper application of this burgeoning statistic.

## 2 The tau statistic

The tau statistic τ is a non-parametric global clustering statistic which takes a disease frequency measure (risk, odds or rate) within a certain annulus around a case and compares it to the background measure (at any distance) and averaged over all cases (Salje et al., 2012, Lessler et al., 2016, Pollington et al., 2019a). It measures the tendency of case pairs to spatially cluster while implicitly accounting for how related they are in terms of transmission using temporal information, making it a *spatiotemporal* statistic.

### 2.1 Tau statistic (odds ratio estimator)

We describe the most common tau estimator ${\overset{ˆ}{\tau }}_{\text{odds}}$, which is based on the relative odds of disease (Lessler et al., 2016), rather than other forms of the statistic (including a new rate ratio estimator), which are described in a detailed review (Pollington et al., 2019a) from which this subsection draws heavily.

**Fig. 1:**
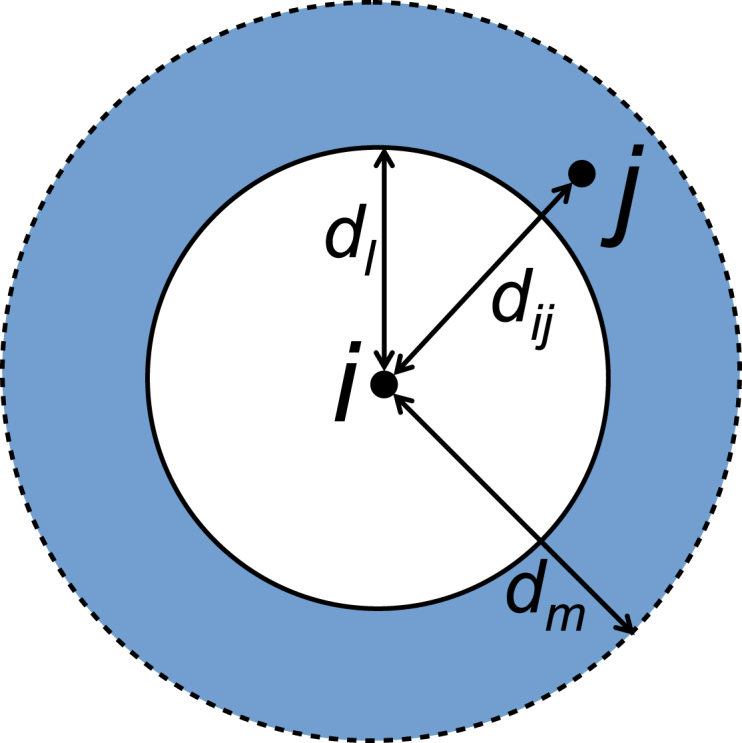
A single distance band $\left[d_{l},d_{m}\right)$, the primary argument of the distance form of a tau estimator function: a half-closed annulus of radii $d_{l},d_{m}$ with a case j inside, around case i, separated by distance $d_{ij}$.

The distance form of the tau statistic $\tau_{\text{odds}}$ is the ratio of (i) the odds $\theta \left(d_{l},d_{m}\right)$ of finding any case j that is ‘related’ to any case i, within a half-closed annulus $\left[d_{l},d_{m}\right)$, ($l,m\in Z^{+}$, m=l+1), around case i, to (ii) the odds θ(0,∞) of finding any case j related to any case i at any distance separation ($d_{ij}\geq 0$) for n total cases (Eq. (1) & Fig. 1).

(1) $${\overset{ˆ}{\tau }}_{\text{odds}}\left(d_{l},d_{m}\right)≔\frac{\overset{ˆ}{\theta }\left(d_{l},d_{m}\right)}{\overset{ˆ}{\theta }\left(0,\infty \right)}$$

$$\text{ where }\overset{ˆ}{\theta }\left(d_{l},d_{m}\right)=\frac{\overset{n}{\underset{i=1}{\sum }}\overset{n}{\underset{j=1,j\neq i}{\sum }}1\left(z_{ij}=1,d_{l}\leq d_{ij}<d_{m}\right)}{\overset{n}{\underset{i=1}{\sum }}\overset{n}{\underset{j=1,j\neq i}{\sum }}1\left(z_{ij}=0,d_{l}\leq d_{ij}<d_{m}\right)}$$

The odds estimate $\overset{ˆ}{\theta }$ in Eq. (1) is the ratio of the number of related case pairs ($z_{ij}=1$) within $\left[d_{l},d_{m}\right)$, versus the number of unrelated case pairs ($z_{ij}=0$) within $\left[d_{l},d_{m}\right)$. The main computation is effectively a double sum over ‘relatedness’ indicator functions 1(⋅) for case pairs.

$\overset{ˆ}{\tau }\left(d_{l},d_{m}\right)$ is then evaluated over B total distance bands to give a *distance band set* $\underline{\Delta }≔\left\{\left[d_{l},d_{m}\right),l,m\in Z^{+},m=l+1,l\leq B\right\}$. Tau values signify either the presence of spatiotemporal clustering (τ>1), no clustering and no inhibition (τ=1) or inhibition (τ<1). Sometimes an expanding disc is described by setting $d_{l}=0$ and relabelling $d_{m}=d$ to give $\overset{ˆ}{\tau }\left(d\right)$ instead. Although $\overset{ˆ}{\tau }$ is strictly evaluated for a given distance band $\left[d_{l},d_{m}\right)$, when a τ-distance graph is drawn a value of $\overset{ˆ}{\tau }\left(d\right)$ can be obtained through linearly interpolating between the distance band endpoints. The half-closed annulus is a correction to the original open interval (Lessler et al. (2016): appendix 5); it was incorporated in December 2018 into the IDSpatialStats *R* package (which calculates the tau statistic) (Giles et al., 2018, Giles et al., 2019).

The relatedness of a case pair $z_{ij}$ is commonly determined using temporal information (*e.g.* difference in onset times of cases i and j, *i.e.* $t_{j}-t_{i}$) (Pollington et al., 2019a). The *serial interval* is the period between the onset times of symptoms in the infector $t_{i}$ and their infectee $t_{j}$. Typically cases are defined as being temporally related when their onset times are within a single mean serial interval of each other.

∗τ∗

In the following sections (Sections 3, 4) we provide a descriptive analysis of the data, before systematically testing several aspects of the tau statistic’s implementation and their impact on the estimated clustering range and its sampling error.

## 3 Methods

### 3.1 The dataset and baseline analysis

We analyse an infectious disease dataset of measles from case households in Hagelloch, Germany in 1861 (Pfeilsticker, 1863, Oesterle, 1992, Neal and Roberts, 2004, Höhle et al., 2019). Computations were coded in *R* (R Core Team, 2019) with further detail in the Supplementary material. We have reproduced Lessler et al.’s (unpublished) analysis as a baseline result (Fig. 3); using *their* interpretation of Fig. 3, spatiotemporal clustering is reported up to ∼30 m (Lessler et al., 2016). As τ is a global statistic, ideally we would explore a distance band set covering the majority of pairwise distances (∼200 m). However here we restrict the plots to 120 m for diagnostic (Fig. 3) or point estimation (Figs. 5, 6, 8 & B.3) purposes, to be consistent with the baseline analysis.

### 3.2 Our approach to graphical hypothesis testing and point estimation

An *envelope* is loosely defined as a collection of connected-line (*syn.* piecewise linear) functions in the Cartesian plane, with some bound applied above and below. *Central*/*null envelopes* describe the line function, *i.e.* whether it originates from bootstrap simulations of a point estimate or *time-mark permuted* null distribution (Section 3.3), respectively; whereas *global envelope* or *pointwise confidence interval* (*syn.* confidence band) refer to the way line functions are bounded. A global envelope is a confidence interval (CI) for a collection of line functions but does not represent a single distance band of one tau point estimate $\overset{ˆ}{\tau }\left(d_{l},d_{m}\right)$ (*i.e.* a pointwise CI), but rather the entire distance band set $\underline{\Delta }$. At say a 95% significance level, in 95% of outcomes of constructing a global envelope, the random envelope would contain the true value of $\tau \left(d_{l},d_{m}\right),\forall \;\left[d_{l},d_{m}\right)\in \underline{\Delta }$ (Baddeley et al., 2015).

**Fig. 2:**
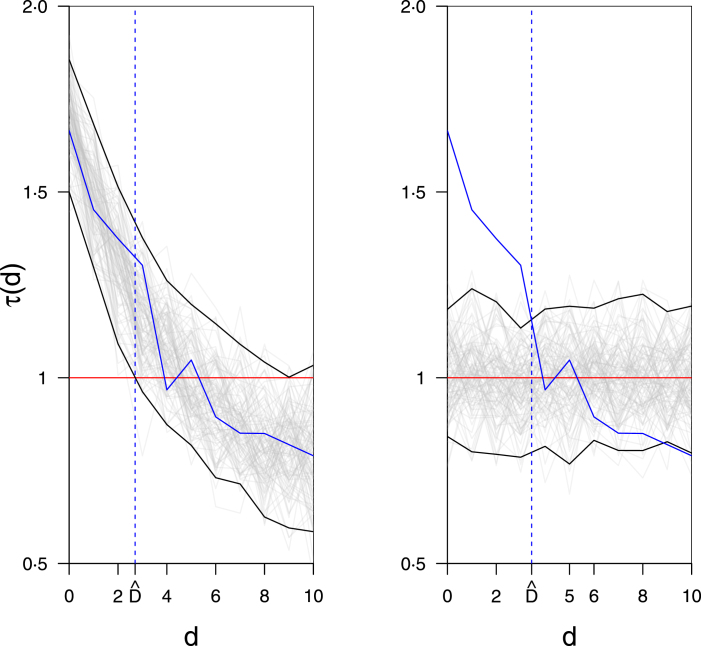
Previous naive methods: several authors (Section 3.2) choose one envelope type as ‘central’ (a) or ‘null’ (b), then simultaneously test the hypothesis of no clustering and estimate the clustering endpoint parameter $\overset{ˆ}{D}$ (Pollington et al., 2019a). The single red line τ=1 represents no spatiotemporal clustering nor inhibition. Grey lines indicate (a) a collection of spatial bootstrap estimates ${\overset{ˆ}{\tau }}^{*}$ (denoted by ${}^{*}$, see Section 3.4.1) from a typical tau estimator characterised by negative exponential lines with Normal noise, or (b) simulations of the tau estimator on time-mark permuted data for null envelope construction, represented here as lines at τ=1 with Normal noise; black lines mark out the envelope bounds constructed from pointwise confidence intervals. The solid blue line characterises an empirical tau point estimate $\overset{ˆ}{\tau }\left(d\right)$. Instead, we split the method into the separate steps of graphical hypothesis testing (Fig. 4) and point estimation (*e.g.* Fig. 5) in Sections 3.3 & 3.4, respectively. (For interpretation of the references to colour in this figure legend, the reader is referred to the web version of this article.)

Our graphical hypothesis test (Section 3.3) and point estimation methods (Section 3.4; online Graphical abstract) offer corrections to the implementations of the tau statistic or similar statistics used in many papers reviewed in Pollington et al. (2019a), *i.e.* (Salje et al., 2012, Salje et al., 2016a, Salje et al., 2016b, Salje et al., 2017, Salje et al., 2018, Bhoomiboonchoo et al., 2014, Grabowski et al., 2014, Levy et al., 2015, Grantz et al., 2016, Hoang Quoc et al., 2016, Lessler et al., 2016, Azman et al., 2018, Rehman et al., 2018, Succo et al., 2018, Truelove et al., 2019). These studies incorrectly used an envelope about the point estimates or simulated null distribution, constructed from pointwise CIs, as a graphical hypothesis test to reject clustering which amounts to multiple hypothesis testing and inflated type I errors (Figs. 2a & b) (Pollington et al., 2019a). Additionally they defined the *clustering endpoint* D as the distance at which the lower bound of the first pointwise (percentile) CI belonging to a point estimate above τ=1, touches τ=1 (Fig. 2a), or where the point estimate line touches the upper bound of the null envelope formed from pointwise CIs (Fig. 2b); thus mixing graphical hypothesis testing with point estimation (Pollington et al., 2019a).

**Fig. 3:**
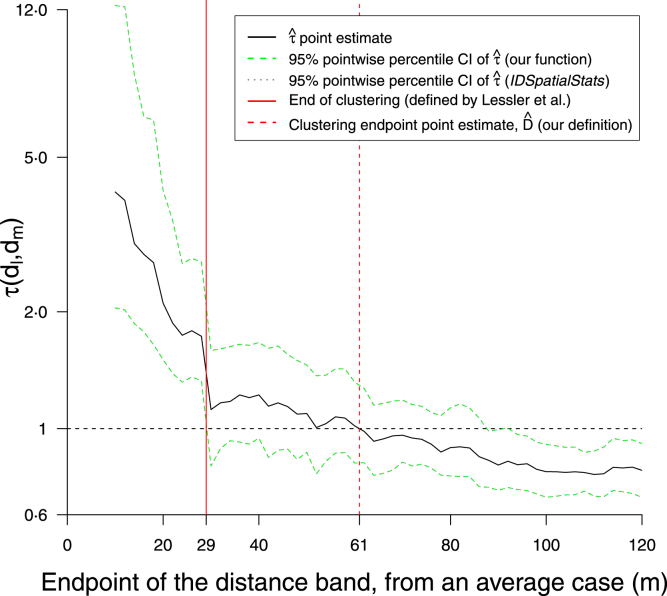
Baseline result: a reproduction of a previous analysis (Lessler et al., 2016 Fig. 4C); note their x-axis used the midpoint of the distance band rather than the endpoint. The superimposition of their envelope and ours validates our implementation of tau functions from their IDSpatialStats*R* package (Giles et al., 2018). According to Lessler et al.’s convention, the end of the clustering range is where the lower bound of the envelope intersects τ=1 (${\overset{ˆ}{D}}_{\text{base}}=29$ m). We do *not* endorse this convention however, and take the point where the point estimate line $\overset{ˆ}{\tau }$ intersects ($\overset{ˆ}{D}=61$ m). Additionally we generally recommend the use of BCa pointwise CIs for diagnostic plots such as this. Their 29 m value was previously reported as 15 m due to a misinterpretation in the midpoint distance graph reading, as confirmed by Lessler (personal comm.)). Instead we use the endpoint so that a point (d,τ(d)) read off the graph pertains to the single distance band $\left[d_{m-1},d_{m}=d\right)$, and more commonly [0,d) when $d_{l}=0$. Whereas the midpoint requires an additional step by the reader that can easily introduce error.

### 3.3 Graphical hypothesis test of no clustering and no inhibition

We instead construct a *global envelope* around the distribution of the null hypothesis ($H_{0}$: τ=1, no spatiotemporal clustering nor inhibition) (Myllymäki and Mrkvička, 2019a). This is generated by randomly permuting the time marks (${\text{onset time}}_{i}$) of the spatiotemporal data points $X_{i}=$ (${\text{x-coordinate}}_{i}$, ${\text{y-coordinate}}_{i}$, ${\text{onset time}}_{i}$), i={1,…,n} to scramble any spatiotemporal clustering present and simulate what $\overset{ˆ}{\tau }$ would be under $H_{0}$. We assess if a subset of distance bands $\underline{\delta }$ of $\underline{\Delta }$ exists (as contiguous or disjoint regions) where the tau point estimate $\overset{ˆ}{\tau }\left(d\right)$ is ever above/below the upper/lower bound, respectively, of this global null envelope. This global envelope is of extreme rank type (“defined as the minimum of pointwise ranks”) with 95% significance level and extreme rank length p-value interval (note: a range, not a single p-value) as constructed by the GET *R* package (Myllymäki et al., 2019b) (see online Graphical abstract). We compute 2500 time-mark permuted tau simulations for an optimal test (Myllymäki et al., 2017).

The test is two-tailed, which is necessary as only once the graph is plotted is the presence of clustering or inhibition apparent (alternative hypothesis $H_{1}$: τ≠1). One should use a *global* $\underline{\Delta }$ covering all pairwise distances, but at large distances null simulations commonly diverge from τ=1. This however can be assessed by tracking the upper & lower quartiles of null simulations (see code in Supplementary material) and choosing a shorter maximum distance; here the maximum pairwise distance is 319 m and we choose 220 m.

### 3.4 Point estimation of the clustering endpoint and its sampling error

If graphical hypothesis testing establishes evidence against no spatiotemporal clustering nor inhibition within a subset of distance bands $\underline{\delta }$ (Section 3.3) and visual graph inspection indicates clustering, then it is acceptable to estimate the *clustering endpoint* $\overset{ˆ}{D}$ for the clustering range $\left[d_{1}=0\text{ (assumed)},d_{m}=\overset{ˆ}{D}\right)$; this is where the point estimate line $\overset{ˆ}{\tau }\left(d\right)$ intercepts τ=1, *i.e.* $\overset{ˆ}{\tau }\left(\overset{ˆ}{D}\right)=1$. Due to discrete distance bands we linearly interpolate between their endpoints, *i.e.* $\left[d_{l},d_{m}\right)$ with $\overset{ˆ}{\tau }>1$, and $\left[d_{l+1},d_{m+1}\right)$ with $\overset{ˆ}{\tau }<1$, to obtain $\overset{ˆ}{D}$.

To estimate the sampling error of $\overset{ˆ}{D}$ we use spatial bootstrap (denoted by ${}^{*}$, see Section 3.4.1) tau estimates ${\overset{ˆ}{\underline{\tau }}}^{*}$. For every bootstrap simulation (that represents a connected line of simulated tau estimates for increasing d, *i.e.* $\left\{{\overset{ˆ}{\tau }}^{*}\left(d_{l},d_{m}\right):\left[d_{l},d_{m}\right)\in \underline{\Delta }\right\}$), we record those that originate from above τ=1 and then intersect τ=1 at some greater distance D within $\underline{\Delta }$, *i.e.* those that satisfy $\left\{{\overset{ˆ}{\tau }}^{*}\left(D\right)=1:D\in \underline{\Delta }\right\}$. We use N=2500 samples which is more than sufficient for a typical bootstrap (Efron and Tibshirani, 1998). We then take this horizontal set of values $\underline{D}$ and use it to obtain a CI to describe the sampling error in $\overset{ˆ}{D}$ (see online Graphical abstract).

We now investigate *spatial* bootstrap methods (Section 3.4.1), CI construction (Section 3.4.2) and distance band sets (Section 3.4.3).

#### 3.4.1 Spatial bootstrap sampling methods for $\overset{ˆ}{\tau }$

The method that generates spatial bootstrap tau estimates ${\overset{ˆ}{\tau }}^{*}$ may impact the sampling error of the CI of the clustering endpoint $\overset{ˆ}{D}$. Through bootstrap theory, the sampling distribution ${\overset{ˆ}{\underline{\tau }}}^{*}$ may serve as a proxy for the actual distribution of $\overset{ˆ}{\tau }$ on the data. Furthermore the (central) envelopes constructed from ${\overset{ˆ}{\underline{\tau }}}^{*}$ may approximate the envelope of $\overset{ˆ}{\tau }$ on the data, and as a corollary the $\underline{D}$ envelope formed by the intercept of ${\overset{ˆ}{\underline{\tau }}}^{*}$ with τ=1 (Efron, 1979).

We compare three *spatial bootstrap* methods that define the bootstrap either on the data beforehand (see *Resampled-index spatial bootstrap* (RISB)), or bootstrap the local τ functions (see *Modified point spatial bootstrap* (MPSB) in Appendix A.4) or locally-(un/)related mark functions m (see *Modified marked point spatial bootstrap* (MMPSB)). All are non-parametric because they randomly resample the data, or local τ or m functions, without imposing a distribution (Loh, 2008).

##### Resampled-index spatial bootstrap (RISB).

We start again with spatiotemporal data set $X={\left(X_{i}\right)}_{i=\left\{1,\ldots ,n\right\}}$ where $X_{i}$ = (${\text{x-coordinate}}_{i}$, ${\text{y-coordinate}}_{i}$, ${\text{onset time}}_{i}$). Using the Uniform distribution we resample with replacement the data’s index vector $\underline{i}=\left(1,\ldots ,n\right)$ n times (equal to the number of cases), to generate a spatial bootstrap of the data, with sample index vector ${\underline{i}}^{*}=\left(i_{1}^{*},\ldots ,i_{n}^{*}\right)$ and subsequent data set $X^{*}=\left(X_{i^{*}}\right)$; $\underline{i}$ and ${\underline{i}}^{*}$ have the same length, but ${\underline{i}}^{*}$ is bound to contain duplicated indices due to sampling with replacement. To obtain N bootstrap τ estimates ${\overset{ˆ}{\underline{\tau }}}^{*}=\left({\overset{ˆ}{\tau }}_{1}^{*},\ldots ,{\overset{ˆ}{\tau }}_{N}^{*}\right)$ we apply the tau odds estimator individually to N bootstrap data sets $X_{1}^{*},\ldots ,X_{N}^{*}$; the same approach could be applied to other τ estimators.

Loh critiques this “naive” sampling with replacement of the points $X_{i}$ of a spatial dataset $\left(X_{i}\right)$ to produce a spatial bootstrap sample, because “the spatial dependence structure has to be preserved as much as possible” (Loh, 2008) … “to reflect properties of the original process” (Loh and Stein, 2004). Lessler et al. (2016) and others used this method and additionally for any $i^{*}$, $j^{*}$ resampled index pairs, dropped pairs $\left(X_{i^{*}},X_{j^{*}}\right)$ for which $X_{i^{*}}=X_{j^{*}}$, as they represented the same point, to avoid ‘self comparisons’.

##### Modified marked point spatial bootstrap (MMPSB).

Like MPSB (discussed in Appendix A.4) we bootstrap local functions not the data (RISB), but like all three methods still use ${\underline{i}}^{*}$ to decide the sample. Unlike MPSB rather than using local τ functions (Equation A.2), we go deeper and compute the number of locally-related/unrelated cases from mark functions $m_{i}\left(k\right)$ local to case i, according to their binary time-relatedness k.

The number of time-related cases (#related) out of all empirical cases j, within a distance $\left[d_{l},d_{m}\right)$ around a case $i^{*}$ (chosen in the bootstrap sample) is:

(2) $$\text{\#related}\left(d_{l},d_{m},k=1,i^{*}\right)\equiv m_{i^{*}}\left(d_{l},d_{m},k=1\right)≔\underset{j\in \underline{j},j\neq i^{*}}{\sum }1\left(d_{l}\leq d_{i^{*}j}<d_{m},z_{i^{*}j}=1\right)$$

and then an average is taken over the n cases in the bootstrap sample of indices ${\underline{i}}^{*}$:

(3) $$\bar{{\text{\#related}}^{*}\left(d_{l},d_{m}\right)}\equiv m^{*}\left(k=1\right)=\frac{1}{n}\underset{i^{*}\in {\underline{i}}^{*}}{\sum }\underset{j\in \underline{j},j\neq i^{*}}{\sum }1\left(d_{l}\leq d_{i^{*}j}<d_{m},z_{i^{*}j}=1\right),$$

and similar steps for time-unrelated cases yield:

(4) $$\bar{{\text{\#unrelated}}^{*}\left(d_{l},d_{m}\right)}\equiv m^{*}\left(k=0\right)=\frac{1}{n}\underset{i^{*}\in {\underline{i}}^{*}}{\sum }\underset{j\in \underline{j},j\neq i^{*}}{\sum }1\left(d_{l}\leq d_{i^{*}j}<d_{m},z_{i^{*}j}=0\right),$$

and finally the odds and odds ratio estimators can be calculated as before:

(5) $$\theta^{*}\left(d_{l},d_{m}\right)=\frac{\bar{{\text{\#related}}^{*}\left(d_{l},d_{m}\right)}}{\bar{{\text{\#unrelated}}^{*}\left(d_{l},d_{m}\right)}}=\frac{\underset{i^{*}\in {\underline{i}}^{*}}{\sum }\underset{j\in \underline{j},j\neq i^{*}}{\sum }1\left(d_{l}\leq d_{i^{*}j}<d_{m},z_{i^{*}j}=1\right)}{\underset{i^{*}\in {\underline{i}}^{*}}{\sum }\underset{j\in \underline{j},j\neq i^{*}}{\sum }1\left(d_{l}\leq d_{i^{*}j}<d_{m},z_{i^{*}j}=0\right)}$$

(6) $$\tau_{\text{MMPSB}}^{*}\left(d_{l},d_{m}\right)=\frac{\theta^{*}\left(d_{l},d_{m}\right)}{\theta^{*}\left(0,\infty \right)}$$

#### 3.4.2 Confidence interval (CI) construction

Bias-corrected and accelerated (BCa) CIs can cope with asymmetrical distributions (like $\underline{D}$ defined in Section 3.4) better than percentile CIs. For non-parametric problems Carpenter and Bithell (2000) consistently found Efron’s BCa method best, due to its low theoretical coverage errors for approximating the exact CI. BCa had “*second-order correct coverage*” errors under some assumptions, while a percentile CI was first-order correct at best (Efron, 1987). The BCa algorithm transforms a distribution of bootstrap calculations by normalisation to stabilise its variance so that a CI can be constructed, then back-transforms it (Efron, 1987). We calculated it using the coxed *R* package (Kropko and Harden, 2019).

#### 3.4.3 Distance band sets

The tau statistic is non-unique as it depends on the distance band set chosen (Pollington et al., 2019a), so the potential variation in τ estimates from this choice is of interest; we explore this briefly in a non-systematic way. From analysing cases’ pairwise distances we propose a reasonable distinct (non-overlapping) distance band set, *i.e.* ${\underline{\Delta }}_{\text{dis}}≔\{$[0,7), [7,15), [15,20), [20,25), [25,30), …, [115,120 m)} as a comparison to the overlapping set in the baseline analysis ${\underline{\Delta }}_{\text{overlap}}≔\{$[0,10), [0,12), [0,14), …, [0,50), [2,52), [4,54), …, [70,120 m)} (Lessler et al., 2016), and test these using N=2500 samples under MMPSB sampling.

## 4 Results & discussion

### 4.1 Dataset description

The epidemic over a small ∼280 x 240 m${}^{2}$ area lasted nearly three months and five distinct generations can be discerned from the epidemic curve (Fig. B.1). Out of the 197 under-14 year olds, 185 became infected, along with three teenagers, leaving 377 remaining teenagers and adults uninfected (Neal and Roberts, 2004). Fig. B.2 indicates a weak signal of direct transmission between cases, as cases with onsets close together in time (shown by similar colours) tended to be nearby to each other.

**Fig. 4:**
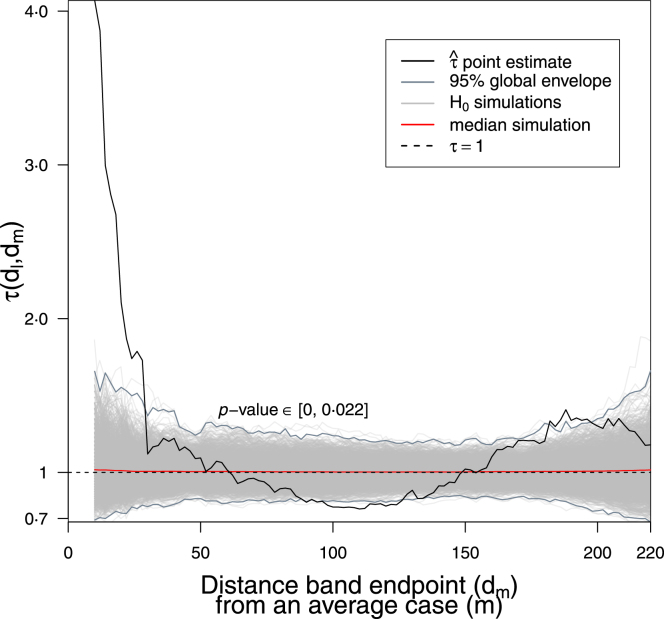
Graphical hypothesis testing: Global envelope test, ‘extreme rank’ type, two-sided at 95% significance level using 2500 simulations of the null hypothesis ($H_{0}$: no spatiotemporal clustering nor inhibition, *i.e.* τ=1), p-value ∈[0,0⋅022]. Note there is a region where $\overset{ˆ}{\tau }$ just exits the global envelope lower bound (suggesting inhibition at ∼100 m) as well as the obvious departures above the upper bound (suggesting clustering at close distances and ∼190 m). We are confident that we are simulating $H_{0}$ well because the median simulation stays close to τ=1 throughout. Distance band set ≔{[0,10),[0,12),[0,14),…,[0,50),[2,52),[4,54),…,[170,220)m}.

### 4.2 Graphical hypothesis test: global envelope *vs.* pointwise CIs

There is moderate evidence against the hypothesis of no spatiotemporal clustering nor inhibition (p-value ∈[0,0⋅022]) based on constructing the global envelope around τ=1 under the null hypothesis (Fig. 4), and thus we conclude that the data X is inconsistent with the null model ($H_{0}$: τ=1). So we turn to the alternative hypothesis, that there is clustering and/or inhibition. Fig. 4 suggests clustering at short distances and ∼190 m, and inhibition at long distances. Unfortunately it is not possible to compare our results with those of previous papers (see Section 3.2), since they used an incorrect pointwise CI approach to assess clustering, for which a p-value is not available.

### 4.3 Impact on the estimated clustering endpoint

**Fig. 5:**
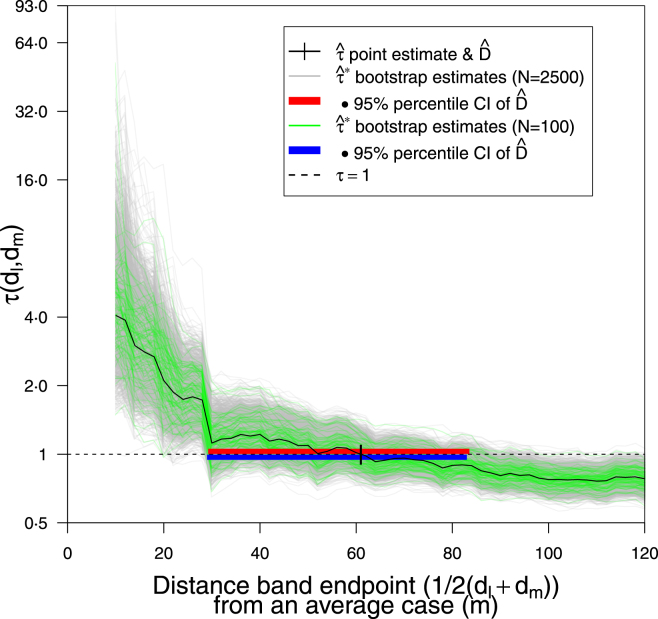
Point estimation: Effect of number of samples on $\overset{ˆ}{D}$ sampling error, when using RISB sampling. Both CIs used 100% of simulations. $\overset{ˆ}{D}=61\text{⋅}0$ m; N=100: 95% percentile CI (29⋅0, 83⋅0 m); N=2500: CI (29⋅2, 83⋅5 m). Distance band set as Fig. 4.

Given the evidence of no clustering with the clear clustering signal in Fig. 4 we continue with parameter calculation. We estimate the clustering endpoint as $\overset{ˆ}{D}=$ 61⋅0 m with a 95% percentile CI of (29⋅0, 83⋅0 m) over 100 bootstrap simulations using RISB sampling (Fig. 5), or (29⋅2, 83⋅5 m) over 2500 simulations (using 100% of simulations, see Appendix A.2); more bootstrap simulations do not appear to affect the sampling error.

The clustering identified at ∼190 m (Fig. 4) is ignored as we are interested in the first clustering range for baseline comparison; in general this may provide useful information of medium-range spatial structure to understand disease spread, but secondary to control policy around an index case household.

**Fig. 6:**
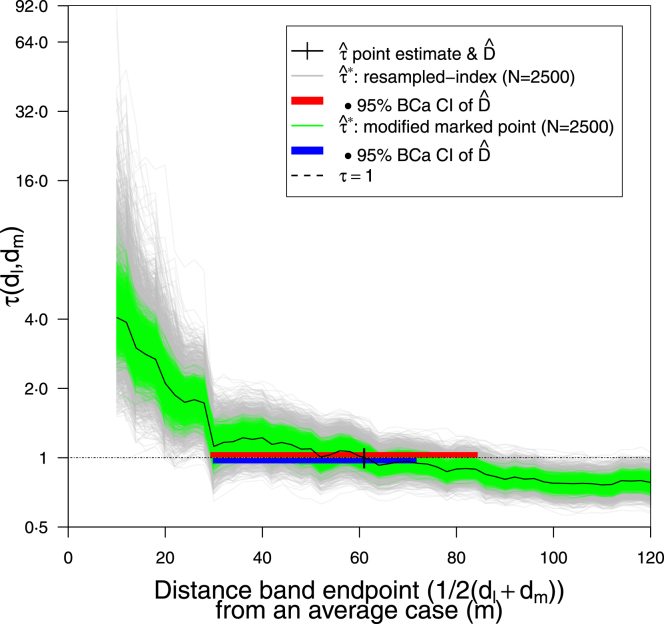
Point estimation: Effect of spatial bootstrap sampling method on $\overset{ˆ}{D}$ sampling error. RISB 95% BCa CI (29⋅3, 84⋅4 m); MMPSB CI (29⋅8, 71⋅8 m); both CIs used 100% of simulations. Distance band set as Fig. 4, $\overset{ˆ}{D}=61\text{⋅}0$ m; N=2500.

 The point estimate $\overset{ˆ}{D}=$ 61⋅0 m is 110% higher than the baseline clustering endpoint (${\overset{ˆ}{D}}_{\text{base}}=29$ m) (Fig. 3). Previous estimates derived via the improper method of finding the distance at which the lower bound of the central envelope (around $\overset{ˆ}{\tau }$) touches τ=1, have probably substantially underestimated this range. The plateauing shape of $\overset{ˆ}{\tau }\left(d\right)$ before it reaches τ=1 contributes to the increased imprecision in the estimate of $\overset{ˆ}{D}$. This highlights the utility of visually assessing the graph rather than rigidly using a τ=1 threshold, as it is likely that disease control over say a 60 m radius around an average case would see the biggest gains over the first 30 m with diminishing returns at wider radii (Fig. 6).

**Fig. 7:**
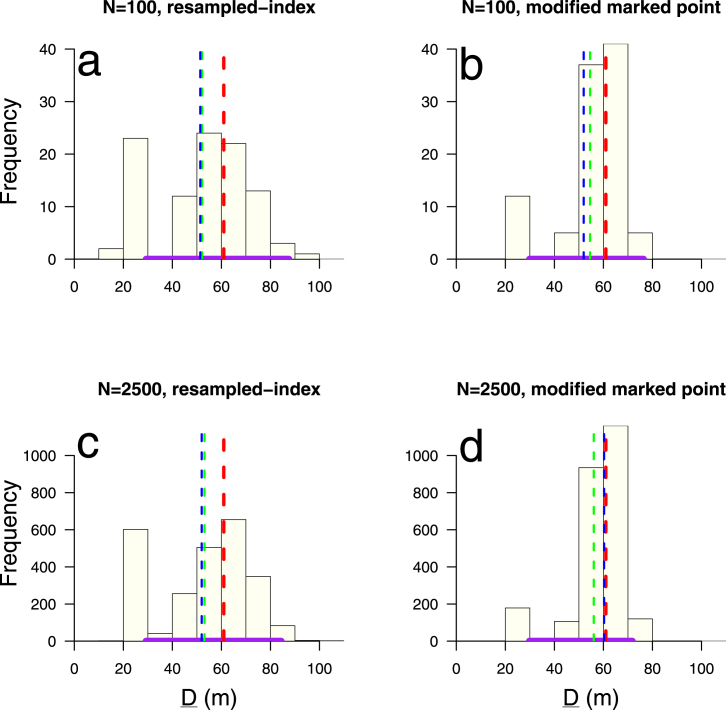
Point estimation: The distribution of $\underline{D}$, formed by the sampled values of ${\overset{ˆ}{D}}_{i}$, *i.e.* $\underline{D}=\left\{{\overset{ˆ}{D}}_{i}:{\overset{ˆ}{\tau }}^{*}\left({\overset{ˆ}{D}}_{i}\right)=1,i=1,\ldots ,N\right\}$ (illustrated in online Graphical abstract), by number of bootstrap samples N = 100 (top row) or N = 2500 (bottom) and by spatial bootstrap sampling method RISB (left column) or MMPSB (right). Vertical dotted lines indicate the point estimate $\overset{ˆ}{D}=61\text{⋅}0$ m (red); with the mean (green) and median (blue) of the sampled estimates ${\overset{ˆ}{D}}_{i}$, obtained from where the bootstrap tau estimates ${\overset{ˆ}{\underline{\tau }}}^{*}$ intercept τ=1. RISB and MMPSB (N=100) have positive skew as the mean estimate is greater than the median estimate, whereas for MMPSB (N=2500) it has a negative skew. All bootstrap estimations have a negative bias with respect to mean or median summary measures versus the point estimate $\overset{ˆ}{D}$. At N=2500 this is ∼10 m for RISB and ∼5 m for MMPSB. The data points used to construct the 95% BCa CIs (purple line on horizontal axis) from the ${\overset{ˆ}{D}}_{i}$ estimates in (a) are copied from Fig. 5 (N = 100 simulations) while those for (c) & (d) are from Fig. 6, while (b) has been freshly calculated. All four CIs used 100% of simulations. Distance band set as Fig. 4. (For interpretation of the references to colour in this figure legend, the reader is referred to the web version of this article.)

**Fig. 8:**
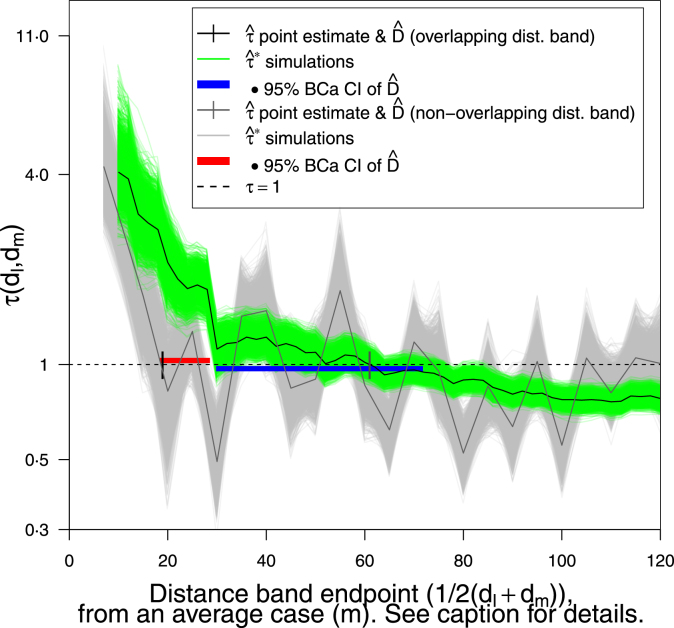
Point estimation: Effect of distance band set on $\overset{ˆ}{D}$ sampling error using MMPSB sampling. Overlapping set ${\underline{\Delta }}_{\text{overlap}}≔\left\{\left[0,10\right),\left[0,12\right),\left[0,14\right),\ldots ,\left[0,50\right),\left[2,52\right),\left[4,54\right),\ldots ,\left[70,120\right)\;\text{m}\right\}$ (Lessler et al., 2016) and distinct ${\underline{\Delta }}_{\text{dis}}≔\left\{\left[0,7\right),\left[7,15\right),\left[15,20\right),\left[20,25\right),\left[25,30\right),\ldots ,\left[115,120\right)\;\text{m}\right\}$. ${\underline{\Delta }}_{\text{dis}}$ yields lower $\overset{ˆ}{D}=18\text{⋅}9$ m and more erratic point estimate $\overset{ˆ}{\tau }$ yet tighter 95% BCa CI for $\overset{ˆ}{D}$ (18⋅4, 28⋅6 m) versus ${\underline{\Delta }}_{\text{overlap}}$ with $\overset{ˆ}{D}=61\text{⋅}0$ m and CI (29⋅8, 71⋅8 m) however on further investigation the distribution $\underline{D}$ for the ${\underline{\Delta }}_{\text{dis}}$ is heavily bimodal; both CIs used 100% of simulations.

The 110% increase in the radial parameter $\overset{ˆ}{D}$ due to the corrected estimation method (Section 3.4) is further compounded for public health interventions, as their time and cost are more closely proportional to area not radius, and the areal increase is 342% (since $\pi \left({\overset{ˆ}{D}}^{2}-{\overset{ˆ}{D}}_{\text{base}}^{2}\right)∕\pi {\overset{ˆ}{D}}_{\text{base}}^{2}=3\text{⋅}42$, assuming $d_{l}=0$).

### 4.4 Spatial bootstrap impact on sampling error: modified marked point *vs.* resampled-index

Using the modified marked point spatial bootstrap (MMPSB) (Section 3.4.1) yields a 24% narrower envelope than the resampled-index spatial bootstrap (RISB), leading to a 95% BCa CI for $\overset{ˆ}{D}$ of (29⋅8, 71⋅8 m) (Fig. 6); both CIs used 100% of simulations.

If through simpler spatiotemporal structures or more evenly spread households the tau point estimate $\overset{ˆ}{\tau }$ had been smoother in approach to the τ=1 intercept, then the range of clustering would be far larger and the benefit of this estimation method (Section 3.4) more apparent. Given the reasons why MMPSB is better (Section 3.4.1), we believe RISB will poorly sample $\overset{ˆ}{D}$ and unfairly represent its precision.

MMPSB outperforms RISB because the latter loses more pair information from resampling indices and avoiding self-comparisons. This was checked empirically for the measles data: the tau point estimate is computed on 188 x 187 = 35156 pairs. In comparison, on average from 1000 simulations, RISB samples from 119 unique people, leading to 119 x 118 = 14042 unique pairs evaluated or ∼39⋅9% of the original pairs. Of course many additional duplicate pairs are used in RISB but we are only interested in unique pair information that is retained. MMPSB only has 119 unique mark functions, but each of them is compared with the other 187 cases, leading to 63⋅3% of pairs being retained.

### 4.5 CI type: BCa *vs.* percentile

Histograms of $\underline{D}=\left\{{\overset{ˆ}{D}}_{i}:{\overset{ˆ}{\tau }}^{*}\left({\overset{ˆ}{D}}_{i}\right)=1,i=1,\ldots ,N\right\}$ by number of bootstrap samples and sampling method strongly indicate asymmetric distributions, which support the principled use of BCa (Figs. 7a-d). Furthermore Figs. 7b & d show how MMPSB tau estimates ${\overset{ˆ}{\underline{\tau }}}^{*}$ intercept at values of d closer to $\overset{ˆ}{D}$ than RISB. Despite this, percentile (29⋅2, 83⋅5 m) and BCa (29⋅3, 84⋅4 m) CIs, using RISB and N=2500, differ marginally (Figs. 5 & 6). RISB appears to introduce a slight positive skew (mean > median) in $\underline{D}$ whereas MMPSB with sufficient samples (N=2500) has a negative skew (Figs. 7a-d). At N=2500, MMPSB noticeably reduces the bias $\left(\underline{\overset{¯}{D}}-\overset{ˆ}{D}\right)$, between mean/median estimates of $\underline{D}$ and the point estimate $\overset{ˆ}{D}$ from ∼10 m to ∼5 m, or ∼8% of $\overset{ˆ}{D}$.

### 4.6 Distance bands: overlapping *vs.* distinct

Overlapping distance band sets ${\underline{\Delta }}_{\text{overlap}}$ appear to produce $\overset{ˆ}{D}$ estimates with higher variance (95% BCa CI (29⋅8, 71⋅8 m)) than distinct ${\underline{\Delta }}_{\text{dis}}$ (CI (18⋅4, 28⋅6 m)) (Fig. 8), but a clearer and smoother trend in tau with increasing distance (both CIs used 100% of simulations). The distribution of $\underline{D}$ is strongly bi-modal for ${\underline{\Delta }}_{\text{dis}}$ because the simulations are more erratic about τ=1. The increased volatility of $\overset{ˆ}{\tau }$ also results in multiple intercepts with τ=1, but for usability we prefer a single range of clustering, given in this case by ${\underline{\Delta }}_{\text{overlap}}$.

## 5 Conclusion and recommendations for improved use

We have shown that the way the endpoint of the clustering range $\overset{ˆ}{D}$ is currently calculated using the tau statistic can lead to biased estimates. Using a redefined $\underline{D}$ for the Hagelloch measles dataset, resulted in bias reductions equivalent to increasing the clustering area of elevated odds by 342%. An improved spatial bootstrap sampling method delivered $\overset{ˆ}{D}$ estimates with 24% lower sampling error. These improvements will appear in future versions of the IDSpatialStats *R* package (Giles et al., 2018). Our results (Section 4) support the following recommendations:

• using the point d where the tau point estimate line $\overset{ˆ}{\tau }\left(d\right)$ intercepts τ=1 to define the clustering endpoint estimate $\overset{ˆ}{D}$ avoids underestimating it like earlier papers. However this estimation should be conditional on graphical hypothesis testing and visual plot inspection.
• the modified marked point spatial bootstrap should be used to simulate $\overset{ˆ}{\tau }$ instead of the resampled-index method, for CIs that better represent the precision of the clustering endpoint $\overset{ˆ}{D}$.
• BCa, rather than percentile, CIs should be used as they give better coverage since the distribution of bootstrap tau simulations ${\overset{ˆ}{\underline{\tau }}}^{*}$ or clustering endpoint estimates $\underline{D}$ is commonly asymmetric.

### Tau statistic limitations.

The distance band set choice $\left[d_{l},d_{m}\right)\in \underline{\Delta }$ clearly biases $\overset{ˆ}{D}$ and affects its smoothness and sampling error. A better understanding of how to choose distance bands for a given purpose is now needed. It is also unknown how the time-relatedness interval choice $\left[T_{1},T_{2}\right]$ (where $z_{ij}=1\left(\left(t_{j}-t_{i}\right)\in \left[T_{1},T_{2}\right]\right)$) biases the tau statistic through inclusion of extraneous co-primary or secondary cases. It is unclear how second-order correlation functions like the tau statistic and Ripley’s K function (Gabriel and Diggle, 2009), originally founded in spatiotemporal point processes with continuous support in $R^{2}$, behave for this spatially discrete process. Finally the number of bootstrap samples required for graphical hypothesis testing and estimation purposes is unknown; we believe that related research by Davidson and MacKinnon (2000) could inform a heuristic algorithm.

∗τ∗

We encourage the adoption of the statistical protocol described (see online Graphical abstract) to properly test for clustering, and, if appropriate, estimate its range. Control programmes have already been informed by the tau statistic and applying these bias-reduction methods will improve its accuracy in future health policy decisions. In addition to modellers or epidemiologists working on real-time outbreaks or post-study analysis, we hope statisticians are inspired to apply this statistic to spatiotemporal branching processes in new fields.

## CRediT contribution statement

**TMP**: Conceptualisation, Methodology, Software, Validation, Formal analysis, Investigation, Data curation, Writing - original draft, Writing - review & editing, Visualisation. **MJT**: Conceptualisation, Writing - review & editing, Supervision. **TDH**: Conceptualisation, Writing - review & editing, Supervision, Funding acquisition. **LACC**: Conceptualisation, Software, Validation, Data curation, Writing - review & editing, Supervision. **Justin Lessler**: Data curation. **Peter J. Diggle**: Methodology, Writing - review & editing.

## Declaration of competing interest

The authors declare that they have no known competing financial interests or personal relationships that could have appeared to influence the work reported in this paper.

## Acknowledgments

TMP would also like to thank Shaun Truelove & John Giles for useful discussions at a poster presentation (Pollington et al., 2019b); Mari Myllymäki for GET *R* package support; and The Editors and Reviewers of *Spatial Statistics* who made pertinent suggestions.

TMP, LACC & TDH gratefully acknowledge funding of the NTD Modelling Consortium by the 10.13039/100000865Bill & Melinda Gates Foundation (BMGF) (grant number OPP1184344) and LACC acknowledges funding of the SPEAK India consortium by 10.13039/100000865BMGF (grant number OPP1183986). Views, opinions, assumptions or any other information set out in this article should not be attributed to BMGF or any person connected with them. TMP’s PhD is supported by the 10.13039/501100000266Engineering & Physical Sciences Research Council, 10.13039/501100000265Medical Research Council and 10.13039/501100000741University of Warwick (grant number EP/L015374/1). TMP thanks Big Data Institute for hosting him during this work. All funders had no role in the study design, collection, analysis, interpretation of data, writing of the report, or decision to submit the manuscript for publication.

## Figure summary

The figures above illustrate the spatial coverage of the statistic (Fig. 1), show previous methods and the baseline analysis (Figs. 2a-b & 3), perform a graphical hypothesis test (Fig. 4), or investigate effects on the parameter point estimate $\overset{ˆ}{D}$ and distribution $\underline{D}$ (unless stated the distance band set is ‘overlapping’, see Fig. 4 caption):

• number of samples N=100 or 2500, on RISB sampling using percentile CIs (Fig. 5)
• RISB *vs.* MMPSB, or MMPSB *vs.* MPSB sampling (Figs. 6 & B.3, respectively) (using N=2500 and BCa CIs)
• RISB *vs.* MMPSB sampling and N=100 or 2500 (with BCa CIs) (Figs. 7a-d)
• overlapping *vs.* distinct distance band sets (using N=2500, MMPSB sampling and BCa CIs) (Fig. 8)

## A Supplementary material

MMC S1 The supplementary material is composed of two appendices. Appendix A details the computation methods and MPBS and MMPBS spatial bootstrap sampling methods. Appendix B contains figures that were not central to the manuscript.
