# Supplementary material for: Developments in statistical inference when assessing spatiotemporal disease clustering with the tau statistic
Source: Spat Stat. 2021 Apr;42:100438. doi: 10.1016/j.spasta.2020.100438 (PMC7985614; doi:10.1016/j.spasta.2020.100438)
Supplement: MMC S1 — The supplementary material is composed of two appendices. Appendix A details the computation methods and MPBS and MMPBS spatial bootstrap sampling methods. Appendix B contains figures that were not central to the manuscript. [file mmc1.pdf]

Appendix A. Extended notes on Methods section

Appendix A.1. Computation methods

The `spatstat` library (1) was used for useful spatial functions, `purrr` for resampling (2), `fields` for image plots (3) and `latex2exp` & `scales` for graph notation (4, 5) and the code of 'January' (2017) for figure labelling. The `IDSpatialStats::get.tau()` and `get.tau.bootstrap()` functions were optimised by re-implementing them in *C* using the *R* packages `Rtools` and `devtools`(7, 8), which sped up τ_{odds} calculations by $\sim$29 times (9) and RStudio IDE (10).

Appendix A.2. Invalidation of the confidence interval for the endpoint of spatiotemporal clustering

The confidence interval (CI) for the endpoint of spatiotemporal clustering $\hat{D}$ is easily invalidated if not all $\hat{\tau}^*$ simulations intersect $\tau = 1$ within the distance band set $\underline{\Delta}$. Caution is needed as the simulations $\underline{D}$ on which the sampling error in $\hat{D}$ is calculated, are not a random sample of the population of simulations $\underline{\hat{\tau}}^*$, which is an important prerequisite for CI construction, as we selectively choose those that cross $\tau = 1$ from above and ignore those that start at or below $\tau = 1$, or above it but never reach $\tau = 1$. Computing CIs at a 95% confidence level on any random sample with a small 5% dropout can substantially decrease the effective confidence level (11). This selection bias is also $\underline{\Delta}$-dependent since if we choose a large enough $\underline{\Delta}$, we may find that simulations that start above $\tau = 1$ eventually do cross $\tau = 1$ and then contribute to the CI. Although we cannot account for this bias, we do report the proportion of simulations used to construct the CIs.

Appendix A.3. Estimating the startpoint of spatiotemporal inhibition

If inhibition was present at greater distances, we ignored estimating its range as it was not of interest. However, if the reader wishes, it can be estimated using a similar algorithm as for estimating clustering. Although one would need to decide from visual analysis of the diagnostic plot if they were monitoring an inhibition range at close distances thus *entering* the global envelope lower bound from below, or *exiting* the lower bound into lower tau values for an inhibition range at moderate distances.

Appendix A.4. Loh & Stein marked point spatial bootstrap (MPSB) applied to the tau odds ratio estimator, not recommended

The Loh & Stein marked point spatial bootstrap (MPSB) is a fast, non-parametric method to obtain a bootstrap distribution of a second-order correlation function (12). For a clustered process simulated by a Matérn process, the CIs constructed using it had a higher empirical coverage than other methods, and computed faster (12).

For the RISB (Section 3.4.1) each bootstrap estimate $\hat{\tau}^*$ is computed from resampled (and smaller) spatiotemporal data X^* containing duplicated points from duplicate indices in $\underline{i}^*$, but the MPSB instead takes a spatial bootstrap sample of the locally-evaluated τ-functions $\underline{\tau}_i$ (Equation A.1) corresponding to each $i^* \in \underline{i}^*$ across all points $\underline{j}, j \neq i^*$, so each local τ_i covers all *points* in X (albeit not all *pairs*) unlike the RISB:

$$\begin{aligned}\hat{\tau}_i(d_l, d_m) &:= \frac{\hat{\theta}_i(d_l, d_m)}{\hat{\theta}_i(0, \infty)} \\ \text{where } \hat{\theta}_i(d_l, d_m) &= \frac{\sum_{j=1, j\neq i}^{n} \mathbb{1}(z_{ij} = 1, d_l \leq d_{ij} < d_m)}{\sum_{j=1, j\neq i}^{n} \mathbb{1}(z_{ij} = 0, d_l \leq d_{ij} < d_m)}\end{aligned} \tag{A.1}$$

The local $\hat{\tau}_i$ functions (Equation A.1) computed for the MPSB are similar to an application of a spatial bootstrap to the K-function (13), which like τ is a second-order correlation function. However we do *not* recommend this literal interpretation of Loh & Stein's method of averaging localised τ-functions for the tau statistic, as the MMPSB method explains (Section 3.4.1 & Appendix A.5), but provide it for completeness (Equation A.2).

$$\tau^*_{\mathrm{MPSB}}(d_l, d_m) = \frac{1}{n}\sum_{i^*} \frac{\theta_{i^*}(d_l, d_m)}{\theta_{i^*}(0, \infty)} = \frac{1}{n}\sum_{i^*} \frac{\left(\frac{m_{i*}(d_l, d_m, k=1)}{m_{i*}(d_l, d_m, k=0)}\right)}{\left(\frac{m_{i*}(k=1)}{m_{i*}(k=0)}\right)} \tag{A.2}$$

Appendix A.5. Advantages and caveats of the modified marked point spatial bootstrap (MMPSB)

The schema (Equations 2-6) is more robust than the original Loh & Stein method (Fig. B.3) when cases i^* have no time-unrelated cases to pair with in their local distance band, i.e. $m_{i^*}(d_l, d_m, k = 0) = 0$ in Equation A.2 causes infinite values for $\theta_{i*}(d_l, d_m)$, or `NaN` values when also $m_{i^*}(d_l, d_m, k = 1) = 0$; the MMPSB simply characterises these null events as zeroes and their addition in Equations 3 & 4 separately protects the rest of the calculation. Alternative remedies to Loh & Stein's approach such as dropping these contributions or merging contiguous distance bands that we attempted proved fruitless—the envelope diverged greatly for short distances and was biased above for larger distances and only 72·6% of simulations contributed to the CI compared to 100% for MMPSB (Fig. B.3). Dropping these inconvenient i^* cases removes important spatial information which the tau bootstrap estimator in Equation A.2 is sensitive to.

MMPSB solves the numerical challenges but is not exactly the Loh & Stein method as we indirectly obtain the tau estimate via calculation of the

spatially bootstrapped odds θ^*, so it is unclear if the validation of their results automatically transfers to our modified form. We also assume the mean of the bootstrap distribution of local mark functions asymptotically approximates the (global) tau statistic, as Loh & Stein only provided experimental evidence to support this (12, 14).

Appendix B. Additional figures

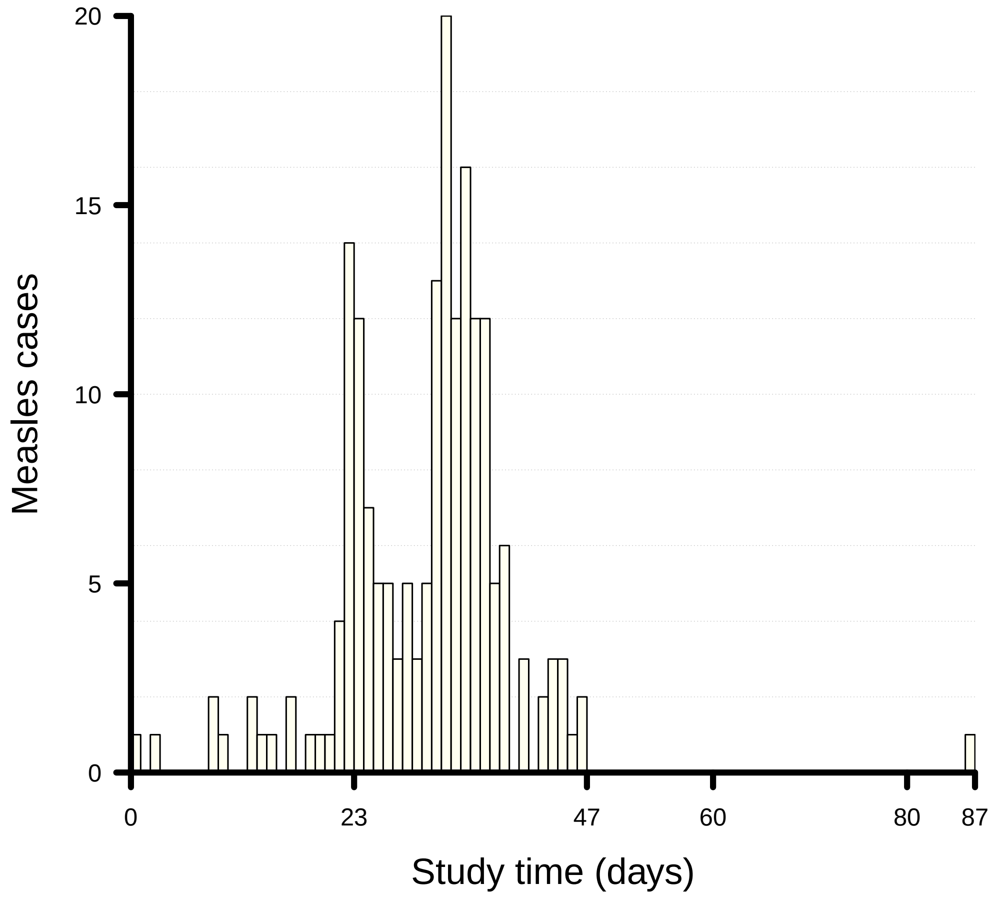

Figure B.1: Epidemic curve of the 188 measles cases in Hagelloch in 1861.

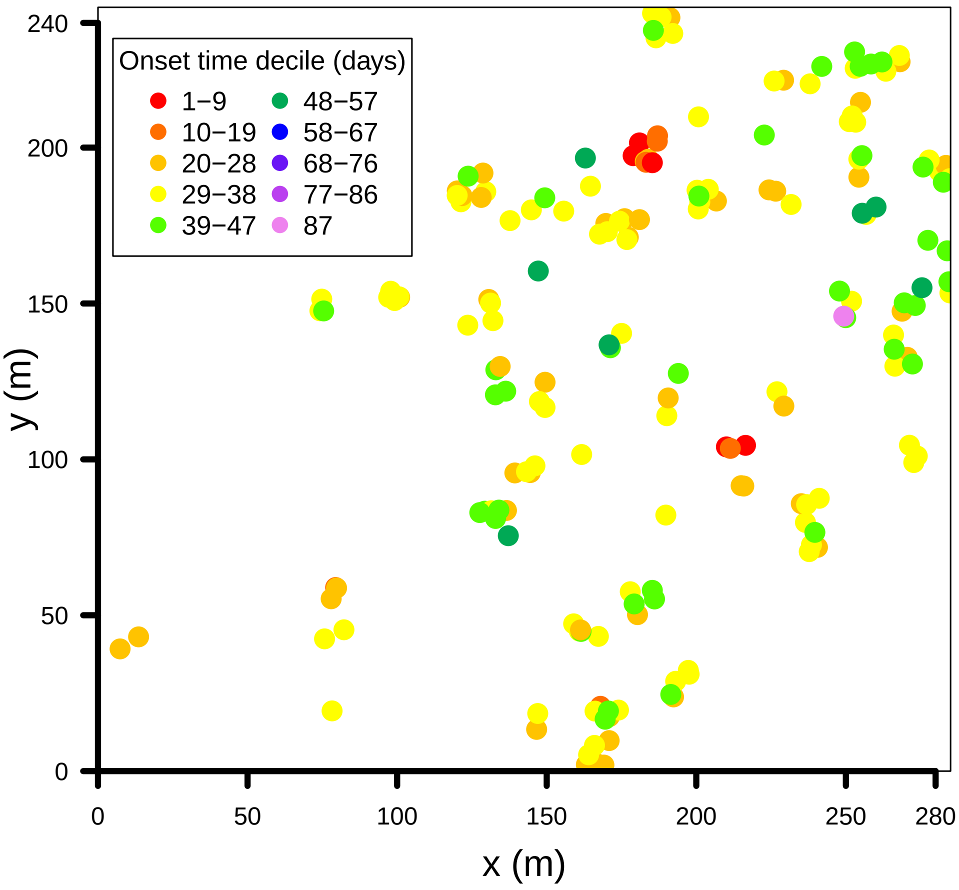

Figure B.2: Spacetime points of cases' locations with onset times as colour marks. Cases jittered up to 5m separately in x and y dimensions using the Uniform distribution to show multiple case households. There is some indication of cases in nearby households (∼50m apart) having a similar date of onset, which may indicate direct transmission up to this distance.

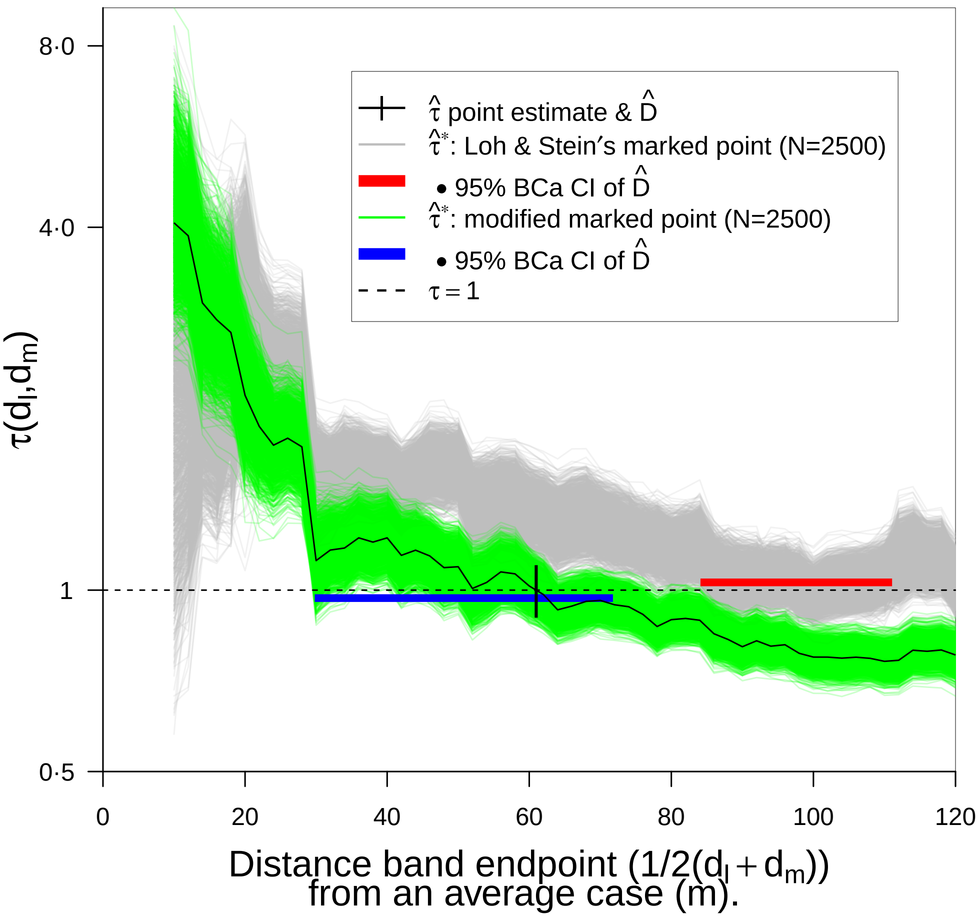

Figure B.3: MMPSB sampling compared with the original Loh & Stein MPSB for the tau statistic. The latter's envelope $\underline{\hat{\tau}}^*$ poorly covers $\hat{\tau}$ at short distances and leads to over-bias in $\hat{\tau}$ at large distances; note that only 72·6% of tau spatial bootstrap simulations $\underline{\hat{\tau}}^*$ contribute to the MPSB BCa CI (84·1, 111·1 m) compared to 100% for MMPSB (29·8, 71·8 m). $\hat{D} = 61{\cdot}0$ m. Distance band set as Fig. 4, $N = 2500$.

References

[1] A. Baddeley, R. Turner, `spatstat` *R* package v1.61-0: An R package for analyzing spatial point patterns, J. Stat. Softw. 12 (6) (2005) 1–42.

[2] L. Henry, H. Wickham, `purrr` *R* package v0.3.3: Functional programming tools, `https://CRAN.R-project.org/package=purrr` (2019).

[3] D. Nychka, R. Furrer, J. Paige, S. Sain, `fields` *R* package v9.9: Tools for spatial data, `https://doi.org/10.5065/D6W957CT` (2017). `doi:https://doi.org/10.5065/D6W957CT`.

[4] S. Meschiari, `latex2exp` *R* package v0.4.0: Use latex expressions in plots, `https://CRAN.R-project.org/package=latex2exp` (2015).

[5] H. Wickham, `scales` *R* package v1.1.0: Scale functions for visualization, `https://CRAN.R-project.org/package=scales` (2018).

[6] January a.k.a user @ztrewq, Adding figure labels (A,B,C,...) in the top left corner of the plotting region, https://logfc.wordpress.com/2017/03/15/adding-figure-labels-a-b-c-in-the-top-left-corner-of-the-plotting-region, accessed: 26/10/2019 (2017).

[7] H. Wickham, J. Hester, W. Chang, devtools: Tools to Make Developing R Packages Easier, *R* package version 2.2.2 (2020).
URL `https://CRAN.R-project.org/package=devtools`

[8] J. Ooms, D. Murdoch, B. Ripley, Rtools: Building R for Windows,

version 3.5 (2020).
URL https://cran.r-project.org/bin/windows/Rtools/

[9] T. M. Pollington, Tau statistic speedup v1.1.1, https://github.com/t-pollington/tau-statistic-speedup (2019). doi:https://doi.org/10.5281/zenodo.3460744.

[10] RStudio Team, Rstudio: Integrated development environment for r v1.2.5001, https://www.rstudio.com (2019).

[11] S. Gorard, Confidence intervals, missing data and imputation: a salutary illustration, Int. J. Res. Educ. Methodol. 5 (3) (2014) 693–698. doi:https://doi.org/10.24297/ijrem.v5i3.3894.

[12] J. M. Loh, M. L. Stein, Bootstrapping a spatial point process, Stat. Sin. 14 (1) (2004) 69–101.
URL https://www.jstor.org/stable/24307180

[13] A. Baddeley, E. Rubak, R. Turner, Spatial Point Patterns: Methodology and Applications with R, 1st Edition, CRC Press/Taylor & Francis, Boca Raton, 2015. doi:https://doi.org/10.1201/b19708.

[14] J. M. Loh, A valid and fast spatial bootstrap for correlation functions, Astrophys. J. (2008) 726–734doi:https://doi.org/10.1086/588631.
